# Feculent Drainage from Percutaneous Endoscopic Gastrostomy Tube due to Gastrocolocutaneous Fistula Found in Emergency Department: A Case Report

**DOI:** 10.5811/cpcem.21286

**Published:** 2024-10-22

**Authors:** Ivan Muchiutti, Emmelyn J. Samones, Tammy Phan, Emily Barrett

**Affiliations:** * Loma Linda University Health School of Medicine, Loma Linda, California; † Loma Linda University Medical Center, Department of Emergency Medicine, Loma Linda, California

**Keywords:** Percutaneous endoscopic gastrostomy, gastrocolocutaneous fistula, PEG replacement, case report

## Abstract

**Introduction:**

Percutaneous endoscopic gastrostomy (PEG) placement is a common procedure for patients requiring non-oral feeding. One rare complication of PEG placement is the formation of a gastrocolocutaneous fistula that develops when the bowel is caught between the stomach and abdominal wall during placement. This report explores an elderly patient’s gastrocolocutaneous fistula development months post-PEG placement who presented with malodorous leakage from the gastrostomy tube to the emergency department (ED).

**Case Report:**

A 73-year-old male on hospice presented to the ED with malodorous leakage from his PEG tube. He had received the PEG tube four months prior to this presentation and had it replaced once at an outside hospital due to blockages. In the ED, his PEG tube was found to have a deflated balloon stopper. The PEG tube was replaced, but the feculent discharge persisted. Imaging showed the tube’s position in the transverse colon. The patient underwent non-surgical management, with PEG tube removal and nutritional support via nasogastric tube. He was discharged with improvement of PEG site.

**Conclusion:**

Gastrocolocutaneous fistula should be considered in patients experiencing unexpected PEG tube drainage or feeding-related complications such as diarrhea. Careful replacement techniques after dislodgement or blockage are important. Radiologic confirmation should be considered after replacement of tubes with feculent drainage. The rarity of gastrocolocutaneous fistula cases in the literature explains the lack of standardized management approaches. Clinical signs such as feculent leakage through the PEG tube site should prompt recognition and diagnosis by the emergency clinician.

## INTRODUCTION

Percutaneous endoscopic gastrostomy (PEG) is one of the most common endoscopic procedures and the gold standard for feeding in patients with viable enteric tracts and difficulty maintaining oral intake.^1^ Like any procedure, PEG placement comes with complications both during placement and much later afterward. Formation of a gastrocolocutaneous fistula is a rare complication of PEG placement that occurs in 0.5% of adults and 3.5% of children.^2^,^3^ Fistula formation is theorized to occur when an interposed segment of small or large bowel is caught between the stomach and abdominal wall during placement and is generally asymptomatic until the tube is dislodged in some manner.^4^,^5^

Patients with a gastrocolocutaneous fistula can stay asymptomatic for months, but when the PEG tube is dislodged either by malfunction or replacement, patients can present with a variety of symptoms including diarrhea after food administration, weight loss, malnutrition, tube blockage, and fecal leakage.^6^,^7^,^8^ Optimal treatment and management has not been clearly determined in the current literature. We present a case of this rare complication in an elderly patient presenting months after initial placement to the emergency department (ED) with foul-smelling leakage from his PEG tube who was ultimately treated with non-surgical management.

## CASE REPORT

A 73-year-old man presented to the ED with a leaking PEG tube with foul-smelling drainage for three days. The patient had a history of Alzheimer dementia and was on hospice at home. He had received a PEG tube four months prior at an outside hospital due to poor oral intake. One month after initial placement, the PEG tube was replaced due to a blockage. The patient improved after PEG replacement and began tolerating oral intake, only requiring tube feeds at night. Three days prior to his visit to the ED, the patient became more lethargic and less interactive. The patient’s family began at this time to note some brown, feculent-appearing leakage coming out from the PEG tube. On physical exam, the patient’s PEG tube had mild leakage at the site and mild skin irritation of the abdominal wall. The existing PEG tube was found to have a deflated balloon. Initially, a bedside exchange of the patient’s PEG tube was performed in the ED. However, after the exchange, persistent foul-smelling feculent drainage was still noted coming from and around the PEG tube. Computed tomography (CT) of the abdomen revealed that “[the] percutaneous gastrostomy tube appears positioned within the transverse colon” and “no bowel dilatation to suggest obstruction” (Image).

The patient was admitted to surgical floor the following day, but the team along with family input opted for medical management. The PEG tube was removed, and a nasogastric tube was placed for nutritional support until the patient began tolerating oral intake again. The patient was discharged to a skilled nursing facility with his PEG tube site healing well.

**CPC-EM Capsule**

What do we already know about this clinical entity?

*Percutaneous endoscopic gastrostomy (PEG) tube placement can result in the formation of a fistula, which can cause diarrhea, weight loss, tube blockage, and leakage.*

What makes this presentation of disease reportable?

*This patient’s fistula was not found during initial tube replacement, but only after replacement of the PEG tube in the emergency department with an atypical presentation.*

What is the major learning point?

*Emergency physicians should have a high index of suspicion for a gastrocolocutaneous fistula in the event of a PEG tube with feculent-appearing drainage.*

How might this improve emergency medicine practice?

*Knowing to consider a fistula in patients with a PEG tube, and this specific constellations of symptoms, can prevent sending patients home with the tube placed incorrectly again.*

## DISCUSSION

Formation of a gastrocolocutaneous fistula is a rare complication of PEG tube placement that is theorized to occur when a decompressed segment of small or large bowel is caught between the stomach and the abdominal wall during tube insertion. The PEG placement is often performed using transillumination via an endoscope.^9^,^10^ The colon becomes interposed between the abdominal wall and the stomach, and the insertion needle then pierces the bowel on its way to the stomach.^10^ If the PEG tube bumper is in the gastric cavity at time of placement, patients are generally asymptomatic until the tube either migrates into the colon from the gastric cavity or is dislodged in some manner.^3^ Figure shows the mechanism of tube slippage secondary to balloon deflation. Replacement of the gastrostomy tube can expose the gastrocolocutaneous fistula if, during replacement, the tube does not follow the fistula all the way into the stomach and is inadvertently placed directly into the colon.^11^ Although most reports describe fistulas forming within the colon, there are reports of jejunocutaneous fistulas as well.^12^

This patient presented four months after initial tube placement and three months after a replacement had been done. The first replacement is suspected to have repenetrated the gastric cavity, but when the balloon bumper deflated at some point shortly before admission, the tube likely slipped back into the colon resulting in the drainage of stool-smelling liquid from the tube. The tube replacement in the ED only managed to enter back into the colon instead of the stomach. Presentation of patients with a gastrocolocutaneous fistula can be secondary to leakage of fecal contents through the PEG tube, as was the case for this patient. Patients may have the opposite problem and present with excessive diarrhea that is often correlated with PEG tube feedings.^6^,^8^,^11^ Other rare presentations can be related to infection, peritonitis, and abscess formation, especially if the tube slips into or is reinserted into the peritoneal cavity and feeds are resumed.^13^ Long asymptomatic periods with a gastrocolocutaneous fistula have been noted, which is suspected to contribute to the lack of exposure to these malfunctions.^5^

Although some case reports describe unexplained migration or slippage of the PEG tube back into the colon, several report the discovery of the gastrocolocutaneous fistula to be shortly after replacement/exchange of the PEG tube^8^,^11^,^12^,^14^ This highlights the importance of correct replacement technique and procedure, especially with the risk of entering the peritoneal cavity and causing further harm. Replacement of the PEG tube is a delicate process as the tract formed with PEG is weaker than one from a surgical gastrostomy.^13^ While no formal guidelines for PEG replacement exists, good control into and along the tract, minimal force with insertion, and the confirmation of proper tube location are principles of a safe replacement.^13^ Confirmation can be done through techniques such as aspirating gastric fluid from the tube or listening for sounds when flushing air through the tube, which can be inconsistent. Radiographically confirming the location of the luminal end of the gastrostomy tube or a contrast study through the replacement tube after placement is a more accurate method that can be considered for patients with suspicious presentations, such as the feculent drainage seen in our patient.^13^

Due to the rarity of these cases in the literature, management has not been standardized. When a gastrocolocutaneous fistula is expected, endoscopy can be used to confirm diagnosis, but radiologic evidence using gastrograffin can be sufficient as well. Management is generally focused on removal of the PEG tube and supportive care for the patient while waiting for spontaneous closure of the fistula. There are, however, cases where endoscopic closure of the fistula was performed.^15^

## CONCLUSION

Gastrocolocutaneous fistula should be considered in patients with feeding difficulties, unexpected PEG tube drainage, or diarrhea with feeds following tube replacement. Replacement of PEG tubes should be done with great care and if questionable, the placement should be confirmed radiologically. Endoscopic diagnosis and management can be considered for complex cases. Emergency physicians should have a high index of suspicion for a gastrocolocutaneous fistula in the event of a PEG tube with feculent-appearing drainage, difficult reinsertion or for a patient presenting with diarrhea following PEG placement.

## Figures and Tables

**Figure:**
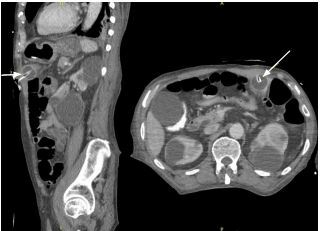
Hypothesized formation of gastrocolocutaneous fistula. (A) Normal percutaneous endoscopic gastrostomy (PEG) insertion (B) Formation of gastrocolocutaneous fistula due to errant PEG insertion. (C) Slippage of PEG tube into colon with deflation of balloon (D). Spontaneous closure of fistula opening in the stomach and PEG tube presence in the colon.^15^ Reproduced with author permission.

**Image:**
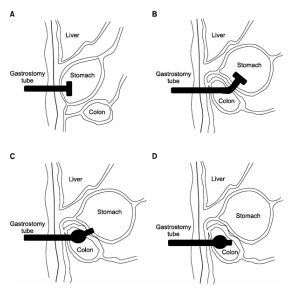
Sagittal (left) and axial (right) views from computed tomography of the abdomen of the percutaneous endoscopic gastrostomy tube positioned and terminating in decompressed transverse colon (arrows).

## References

[1] Rahnemai-Azar AA, Rahnemaiazar AA, Naghshizadian R (2014). Percutaneous endoscopic gastrostomy: indications, technique, complications and management. World J Gastroenterol.

[2] Pitsinis V, Roberts P (2003). Gastrocolic fistula as a complication of percutaneous endoscopic gastrostomy. Eur J Clin Nutr.

[3] Patwardhan N, McHugh K, Drake D (2004). Gastroenteric fistula complicating percutaneous endoscopic gastrostomy. J Pediatr Surg.

[4] Cheung SW (2016). A silent and chronic complication of percutaneous endoscopic gastrostomy tube: small bowel enterocutaneous fistula. Case Rep Gastrointest Med.

[5] Kim HS, Bang CS, Kim YS (2014). Two cases of gastrocolocutaneous fistula with a long asymptomatic period after percutaneous endoscopic gastrostomy. intest Res.

[6] Lee J, Kim J, Kim HI (2018). Gastrocolocutaneous fistula: an unusual case of gastrostomy tube malfunction with diarrhea. Clin Endosc.

[7] Vidal DV, Plaza FJ, David VV (2022). Misplacement of the PEG tube through the transverse colon, an uncommon but possible complication. Rev Esp Enferm Dig.

[8] Okutani D, Kotani K, Makihara S (2008). A case of gastrocolocutaneous fistula as a complication of percutaneous endoscopic gastrostomy. Acta Medica Okayama.

[9] Lenzen H, Weismüller T, Bredt M (2012). Gastrointestinal: PEG feeding tube migration into the colon; a late manifestation. J Gastroenterol Hepatol.

[10] Najafi K, Markowski H, Green D (2023). Managing a gastrocolocutaneous fistula with delayed presentation after PEG placement without surgery. J Case Rep.

[11] Jamma S, Tippor S, Reddy JA (2007). Colocutaneous fistula causing diarrhea: a complication of PEG tube replacement: 420. Am J Gastroenterol.

[12] Karhadkar AS, Schwartz HJ, Dutta SK (2006). Jejunocutaneous fistula manifesting as chronic diarrhea after PEG tube replacement. J Clin Gastroenterol.

[13] Lohsiriwat V (2013). Percutaneous endoscopic gastrostomy tube replacement: a simple procedure?. World J Gastrointest Endosc.

[14] Marcy P, Magne N, Lacroix J (2004). Late presentation of a gastrocolic fistula after percutaneous fluoroscopic gastrostomy. JBR BTR.

[15] Jung TY, Lee JR, Seok DK (2013). Endoscopic management for colocutaneous fistula as a complication of percutaneous endoscopic gastrostomy. Korean J Helicobacter Up Gastrointest Res.

